# Altered brain connectivity in 3-to 7-year-old children with autism spectrum disorder^☆^^☆☆^

**DOI:** 10.1016/j.nicl.2013.03.003

**Published:** 2013-03-19

**Authors:** Mitsuru Kikuchi, Kiyomi Shitamichi, Yuko Yoshimura, Sanae Ueno, Hirotoshi Hiraishi, Tetsu Hirosawa, Toshio Munesue, Hideo Nakatani, Tsunehisa Tsubokawa, Yasuhiro Haruta, Manabu Oi, Yo Niida, Gerard B. Remijn, Tsutomu Takahashi, Michio Suzuki, Haruhiro Higashida, Yoshio Minabe

**Affiliations:** a Research Center for Child Mental Development, Kanazawa University, Kanazawa, Japan; b Department of Psychiatry and Neurobiology, Graduate School of Medical Science, Kanazawa University, Kanazawa, Japan; c Higher Brain Functions & Autism Research, Department of Child Development, United Graduate School of Child Development, Osaka University, Kanazawa University and Hamamatsu University School of Medicine, Osaka University, Osaka, Japan; d International Education Center, Kyushu University, Fukuoka, Japan; e Department of Anesthesiology, Graduate School of Medical Science, Kanazawa University, Kanazawa, Japan; f Department of MEG, Yokogawa Electric Corporation, Tokyo, Japan; g Department of Neuropsychiatry, University of Toyama, Toyama, Japan

**Keywords:** Autism spectrum disorder (ASD), Connectivity, Laterality index, Magnetoencephalography (MEG), Young children

## Abstract

Autism spectrum disorder (ASD) is often described as a disorder of aberrant neural connectivity and/or aberrant hemispheric lateralization. Although it is important to study the pathophysiology of the developing ASD cortex, the physiological connectivity of the brain in young children with ASD under conscious conditions has not yet been described. Magnetoencephalography (MEG) is a noninvasive brain imaging technique that is practical for use in young children. MEG produces a reference-free signal and is, therefore, an ideal tool for computing the coherence between two distant cortical rhythms. Using a custom child-sized MEG, we recently reported that 5- to 7-year-old children with ASD (n = 26) have inherently different neural pathways than typically developing (TD) children that contribute to their relatively preserved performance of visual tasks. In this study, we performed non-invasive measurements of the brain activity of 70 young children (3–7 years old, of which 18 were aged 3-4 years), a sample consisting of 35 ASD children and 35 TD children. Physiological connectivity and the laterality of physiological connectivity were assessed using intrahemispheric coherence for 9 frequency bands. As a result, significant rightward connectivity between the parietotemporal areas, via gamma band oscillations, was found in the ASD group. As we obtained the non-invasive measurements using a custom child-sized MEG, this is the first study to demonstrate a rightward-lateralized neurophysiological network in conscious young children (including children aged 3–4 years) with ASD.

## Highlights

• A custom child-sized magnetoencephalography system was used for young children.
• Rightward intrahemispheric connectivity was observed in autism spectrum disorder.
• Rightward connectivity via gamma band was observed in autism spectrum disorder.

## 1 Introduction

Autism spectrum disorders (ASD) appear in infancy or early childhood, causing delays or impairments in social interaction, communication, and a restricted range of interests. With recent developments in neuroimaging methods, aberrant brain connectivity in ASD has been demonstrated by determining affected functional connectivity (Anderson et al., 2011; Coben et al., 2008; Just et al., 2004, 2007; Kana et al., 2006, 2007; Kleinhans et al., 2008; Koshino et al., 2005; Lai et al., 2010; Lee et al., 2009; Mizuno et al., 2006; Monk et al., 2009; Murias et al., 2007; Noonan et al., 2009; Shih et al., 2010, 2011; Tsiaras et al., 2011; Villalobos et al., 2005; Welchew et al., 2005). While a number of previous studies have reported underconnectivity in the ASD cortex (Coben et al., 2008; Just et al., 2004, 2007; Kana et al., 2006, 2007; Villalobos et al., 2005), some recent studies have indicated mixed or overconnectivity (Lee et al., 2009; Mizuno et al., 2006; Monk et al., 2009; Muller et al., 2011; Noonan et al., 2009; Shih et al., 2010, 2011) and/or aberrant lateralization in ASD brain connectivity (Koshino et al., 2005; Lee et al., 2009; Monk et al., 2009). The accumulated evidence suggests that both the aberrant brain connectivity and the aberrant lateralization reflect important aspects of network dysfunction associated with the pathophysiology of ASD. However, no previous studies have focused on functional brain connectivity in younger ASD children (e.g. < 6 years old) under conscious conditions, with the exception of our recent study on 5- to 7-year-old children with ASD using a custom child-sized magnetoencephalogram (MEG) (Kikuchi et al., 2013).

**Fig. 1:**
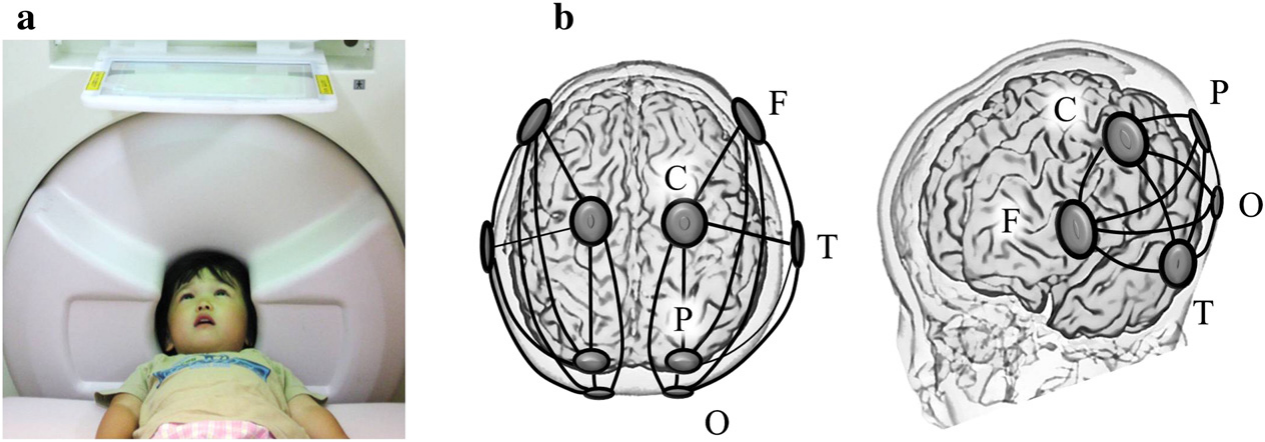
(a), In the custom child-sized MEG system, the MEG sensors are positioned as close to the whole head as possible for optimal recording in young children. During the MEG recording, the children lay supine on a bed and viewed video programs projected onto a screen. (b), Schema of 5 selected sensors and 10 connections of interest (solid lines) in each hemisphere. F, selected sensor in the frontal area; C, central; P, parietal; O, occipital; T, temporal.

Here, we investigated neurophysiological connectivity under conscious conditions in young children, including in children aged 3–4 years, who were either typically developing (TD) or were diagnosed with ASD, using a custom child-sized MEG. This is a useful technique for analyzing young children (Fig. 1a) that can provide measures of cortical neural activity on a millisecond timescale. An assessment of coherence can determine the degree of phase-locking between the activities recorded by different sensors in MEG studies. High coherence between two MEG signals reflects phase-locked neuronal oscillations, suggesting functional integration between neural populations, whereas low coherence suggests independently active populations, suggesting functional segregation. MEG produces a reference-free signal and is therefore an ideal tool for computing coherence between two distant cortical rhythms. In the custom child-sized MEG system used in the present study, the MEG sensors were positioned as close to the whole head as possible for optimal recording, even in the young children (Kikuchi et al., 2010, 2011), which would have been difficult to accomplish with a conventional adult-sized MEG system.

We postulated that young children with ASD have aberrant brain connectivity and/or aberrant lateralization in their brain connectivity. In addition, converging evidence from recent EEG and MEG studies suggests that the properties of gamma oscillations are altered in ASD during information processing (Brown et al., 2005; Grice et al., 2001; Orekhova et al., 2007; Perez Velazquez et al., 2009; Stroganova et al., 2012; Sun et al., 2012; Wilson et al., 2007; Wright et al., 2012). In children with ASD (aged 3 to 8 years), a previous EEG study demonstrated that excess gamma band oscillations during sustained visual attention are directly related to the degree of developmental delay (Orekhova et al., 2007). Therefore, we also investigated whether young children with ASD have increased gamma band power, which is related to the degree of developmental delay.

## 2 Material and methods

### 2.1 Subjects

The clinical group included 35 children with autism spectrum disorder (29 males, 6 females), aged 40–93 months, who were recruited from Kanazawa University’s Hospital and the prefectural hospitals in Toyama. The children were diagnosed by a clinical psychiatrist and a clinical psychologist with over 5 years of experience in ASD using the Autism Diagnostic Observational Schedule, Generic (ADOS) (Lord et al., 1999), the Diagnostic Interview for Social and Communication Disorders (DISCO) (Wing et al., 2002), the DSM-IV (Association, 1994) criteria at the time of MEG, and the Kaufman Assessment Battery for Children (K-ABC) (Kaufman and Kaufman, 1983) for data acquisition. All the ASD children included in this study fulfilled the diagnosis of childhood autism (n = 25), atypical autism (n = 4) or Asperger’s syndrome (n = 6) with DISCO. Children below the ADOS cut-offs were included in the present study if they met the criteria for ASD using both the DSM-IV and DISCO (5 out of 35 children). The control subjects were 35 typically developing children (29 males, 6 females), aged 39–85 months, with no reported behavioral or language problems. The control children were approximately age-matched to the subjects with autism.

All the typically developing children were native Japanese and had no previous or existing developmental, learning, or behavioral problems according to information obtained from a questionnaire completed by their parents. All the participants had normal hearing ability according to the available medical records. Dominant hands, based on preference when handling a spoon, were the following: TD children (right = 34, left = 1, both = 0), ASD children (right = 26, left = 3, both = 6). As shown in Table 1, the two groups were closely matched in chronological age and were approximately matched in their scores on the K-ABC mental processing scale. Parents agreed to their child’s participation in the study with full knowledge of the experimental nature of the research, and written informed consent was obtained prior to participation. The Ethics Committee of Kanazawa University Hospital approved the methods, and all the procedures were performed in accordance with the Declaration of Helsinki.

### 2.2 Recordings and analysis

Recordings and offline analysis of the MEG data were performed as described in our previous study (Kikuchi et al., 2011). All the children participated in the cognitive tasks and MEG measurements on two separate days. On the first day, the participants were subjected to the cognitive tests and were introduced to the environment of MEG measurement. On the second day, the participants were given instruction regarding the MEG measurement. The MEG data were recorded with a multichannel SQUID (Super conducting Quantum Interference Device) whole-head coaxial gradiometer MEG system for children (PQ 1151R; Yokogawa/KIT, Kanazawa, Japan) in a magnetically shielded room (Daido Steel, Nagoya, Japan). The MEG data were acquired with a sampling rate of 1000 Hz and were filtered with a 200 Hz low-pass filter. During the MEG recording, the children lay supine on a bed and viewed a video program projected onto a screen. During the MEG recording time, we determined the position of the head within the helmet by measuring the magnetic fields created after passing current through 3 coils attached to the head surface as fiduciary points, with respect to the bilateral mastoid processes and nasion landmarks.

Prior to recording, we prepared a number of video programs with stories that were especially attractive to young children. Selection of a video program was based on the preference of each participant. The sound of the narration was carried to participants binaurally from electric speakers placed outside the shielded room through a tube placed in front of the subject. Before recording, we asked the children to confirm whether they were content with the video program. During MEG recording, one staff member (author Y.Y.) remained in the shielded room to confirm that each participant was concentrating on the video program and to encourage the participants to maintain a steady body position when necessary.

Offline analysis of the MEG data was performed using Brain Vision analyzer (Brain Products GmbH, Gilching, Germany) and Matlab (MathWorks, Natick, MA). The MEG data were resampled at 500 Hz. Data was segmented for 2.5 s. The selection of artifact-free segments was based on visual inspection. Segments containing head movements, blinks, or muscle activity were carefully excluded from the analysis. The process of eliminating contaminated data was performed blind to personal data. A minimum 37.7 s recording period (i.e., 15 epochs) was accepted for each subject. The average number of available epochs per subject was 58.8 ± 11.0 (mean ± standard deviation) and 54.1 ± 14.6 (mean ± standard deviation) for the TD and ASD children, respectively. MEG spectra were calculated using a fast Fourier transform (FFT) with a spectral resolution of 0.244 Hz.

As in our previous study (Kikuchi et al., 2011, 2013), we focused on the following 5 regions of interest: frontal (F), central (C), temporal (T), parietal (P) and occipital (O) (Fig. 1b). Prior to the offline analysis, selection of sensors corresponding to these brain areas was performed based on the following algorithm. First, using anatomical MRI brain images from 3 young children, we calculated the distances between the sensors and the hippocampus or posterior cingulate cortex (PCC) for 151 sensors. Then, in each hemisphere, we identified the sensors that were closest to the hippocampus or PCC, which were located close to the T and P areas, respectively.

Next, we selected sensors corresponding to the F, C and O areas based on the following requirements: (i) inter-sensor distances between T and F, T and C or T and O that were similar to those between T and P (i.e., within a range of 100 to 120 mm); (ii) the sensor corresponding to the F area was selected from the front row of sensors of the MEG device; (iii) the sensor corresponding to the C area was selected from a row of sensors midway between the sensors corresponding to the F and P areas; and (iv) the sensor corresponding to the O area was selected so that the sensor corresponding to the P area was located midway between the sensors corresponding to the C and O areas. Finally, the sensors corresponding to the F and O areas were located close to the dorsal prefrontal area and occipital area, respectively.

Ten intrahemispheric coherences for each hemisphere were measured between these 5 regions of interest. Coherences (Cross-Spectrum/Autospectrum) were calculated following a Fourier transform using the formula: Coherence (c1, c2)(f) = | CS(c1, c2)(f) |^2^/(| CS(c1, c1)(f) | | CS(c2, c2)(f) |), in conjunction with CS(c1, c2)(f) = Σ c1, i (f) c2, i (f)* (CS, Cross-Spectrum). In the second formula, totaling was carried out via segment number i. Calculation of the average also relates to segments with a fixed frequency, f, and a fixed channel, c. Using this methodology, values between 0 and 1 were obtained for each frequency and for each channel.

Coherences and relative power values were grouped into the following 8 bands, according to our previous study on TD young children (Kikuchi et al., 2011, 2013): delta (0.7–3.9 Hz), theta-1 (4.2–5.9 Hz), theta-2 (6.4–7.8 Hz), alpha-1 (8.3–9.8 Hz), alpha-2 (10.0–12.0 Hz), beta-1 (12.2–19.8 Hz), beta-2 (20.0–29.8 Hz), gamma-1 (30.0–57.9 Hz), and gamma-2 (62.0–79.8 Hz). Laterality indices (LIs) were calculated for each coherence value using the formula LI = (L − R)/(L + R). Therefore, positive values of LI denote more leftward lateralized coherence values and negative values denote less leftward (or more rightward) lateralized coherence values.

### 2.3 Statistics

For the z-transformed coherences, LIs and natural log transformed relative powers, unpaired *t* tests were performed between the ASD and TD groups. Due to multiple comparisons between the 10 intrahemispheric pairs in each hemisphere for 9 frequency bands, our alpha level was adjusted to 0.05/90 = 0.00056 (*t* values > 3.62 or < − 3.62) for intrahemispheric coherence and LI. Due to multiple comparisons between the 5 relative powers in each hemisphere for 9 frequency bands, our alpha level was adjusted to 0.05/45 = 0.00111 (*t* values > 3.41 or < − 3.41) for the relative power values. As a complementary approach, an alpha level of 0.05 was also employed at the risk of a Type I error to explore differences in the physiological measures between the ASD and TD groups.

## 3 Results

### 3.1 Intrahemispheric coherence

**Fig. 2:**
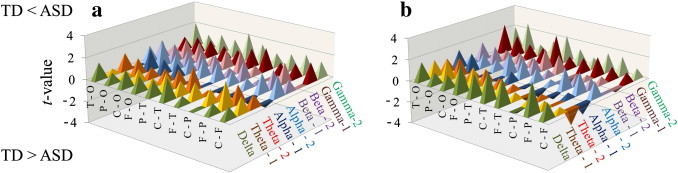
(a, b), The *t* values of intrahemispheric coherence for the left (a) and right (b) hemispheres between the ASD (*n* = 35) and TD (*n* = 35) children. There were no significant differences in the intrahemispheric coherences of either hemisphere between the ASD and TD children (*P* > 0.00056). ASD, autism spectrum disorder; TD, typically developing; F, frontal; C, central; P, parietal; O, occipital; T, temporal.

There were no significant differences in the intrahemispheric coherences between the ASD and TD children (Fig. 2a, b).

### 3.2 Laterality index of intrahemispheric coherence

**Fig. 3:**
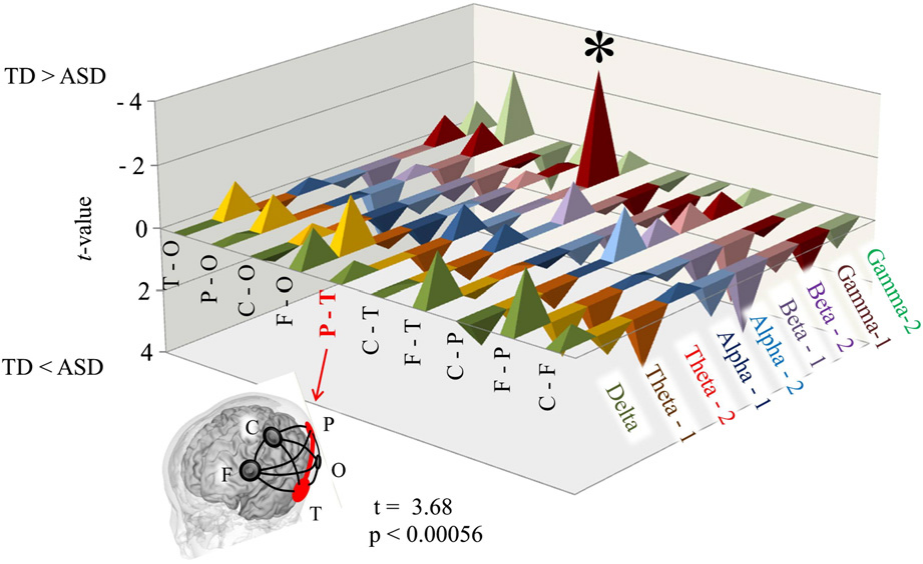
*t* values of the LIs for intrahemispheric coherence between the ASD (*n* = 35) and TD (*n* = 35) children. Note that significantly lower left lateralization was found in the ASD children compared to the TD children, in parietotemporal connectivity in gamma-1 (*t* = − 3.63, *P* < 0.00056). LI, laterality index; ASD, autism spectrum disorder; TD, typically developed; F, frontal; C, central; P, parietal; O, occipital; T, temporal.

**Fig. 4:**
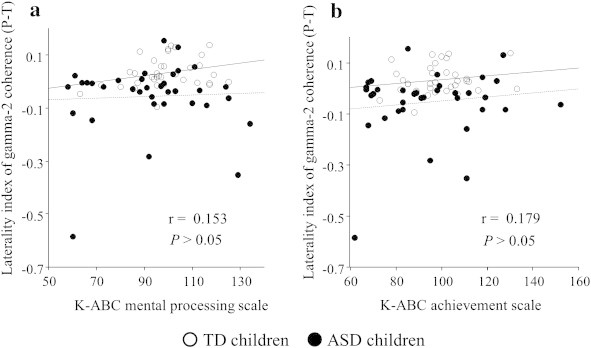
Scatter plot of the laterality index of gamma-1 band coherence (parietotemporal connectivity), K-ABC mental processing scale (a) (n = 70, r = 0.153, P > 0.05), and K-ABC achievement scale (b) (n = 70, r = 0.179, P > 0.05). ○, TD children (n = 35); ●, ASD children (n = 35). L, left; R, right; ASD, autism spectrum disorder; TD, typically developing; Solid line, regression line for TD children; Broken line, regression line for ASD children. There was no effect of a general cognitive quotient on the significant difference between two groups in the LI of the gamma-1 band coherence (P–T).

An unpaired *t* test demonstrated a significantly lower LI of the gamma-1 band coherence (parietotemporal network) in children with ASD compared to TD children (*t* = –3.63, *P* < 0.00056) (Fig. 3). To evaluate the existence of a possible effect of a general cognitive quotient on the significant difference between the two groups in the LI of the gamma-1 band coherence (P–T), we calculated Pearson's correlation coefficients to evaluate the relationships between this LI and two types of general cognitive quotients on the K-ABC (normalized with age) for all subjects (n = 70). The significance level was set at *P* < 0.05. As shown in Fig. 4, this LI showed no significant correlation with the K-ABC mental processing scale (r = 0.153, *P* > 0.05) or with the K-ABC achievement scale (r = 0.179, *P* > 0.05). Even if we investigated these correlations separately for each group (n = 35), the Pearson’s correlation coefficients failed to reveal any significant correlations between these values.

### 3.3 Relative power value

There was no significant difference in the relative power value between the ASD and TD children.

### 3.4 A complementary approach (alpha level of 0.05)

With an alpha level of 0.05, at the risk of a Type I error, some significant differences arose between the two groups. A number of significantly higher intrahemispheric coherences emerged in the ASD group, which exceeded the number expected by chance (18 significant cases in 180 comparisons). However, the number of significant differences remained at chance for the LIs of intrahemispheric coherence (4 significant cases in 90 comparisons) and relative power (5 significant cases in 90 comparisons).

### 3.5 A complementary approach (LI from the full set of MEG sensors)

As a supplementary analysis, we performed an additional analysis of coherence using the full set of MEG sensors, i.e., 151 channels. Sensors that corresponded to the right P–T, in which significant differences were observed in the IL, were selectively used as seed sensors. For each hemisphere, we then calculated the coherence values between the seed sensor (corresponding to P or T) and the remaining 81 sensors (70 sensors in the same hemisphere and 11 sensors on the midline) in the gamma-1 band.

**Fig. 5:**
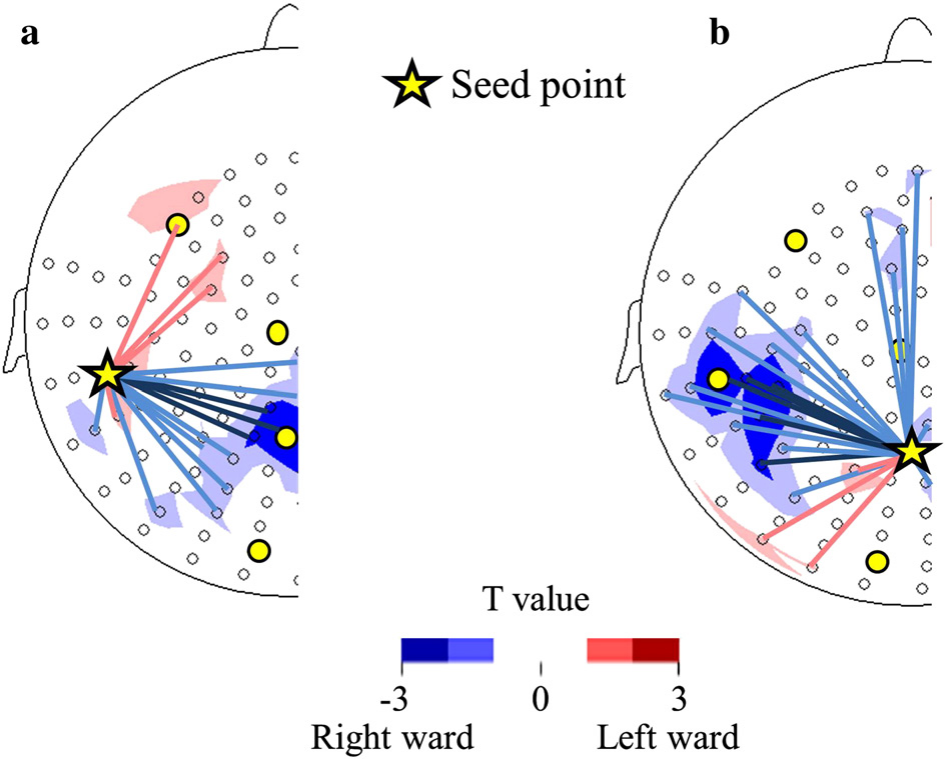
The *t* values of the LIs for the intra-hemispheric coherences in the gamma-1 band between the ASD (n = 35) and TD (n = 35) children are topographically indicated. For each hemisphere, we calculated the coherence values between the seed sensor (corresponding to P or T) and the remaining 81 sensors (70 sensors in the same hemisphere and 11 sensors on the midline) in the gamma-1 band and calculated the LIs for each coherence value. (a) A seed sensor was selected in the temporal region. The t value indicated less left lateralized coherence between the temporal sensor (seed sensor) and the sensors in the parietal regions in children with ASD compared to the TD children. (b) A seed sensor was selected in the parietal region. The t value indicated less left lateralized coherence between the parietal sensor (seed sensor) and the sensors in the temporal regions in children with ASD compared to TD children. The yellow star indicates the seed sensor. The yellow circles indicate the sparse alignment of the MEG sensors for the first analysis in the present study. The open circles indicate the remaining 150 sensors. Red areas (or lines) mean more left lateralized coherence areas and blue areas (or lines) mean less left (or more right) lateralized coherence areas in children with ASD compared to TD children.

We used an unpaired *t* test to reveal specific, lateralized intra-hemispheric connectivity via gamma-1 oscillation that was significantly different between the ASD children and the TD children. As shown in Fig. 5a, when we used the temporal region as a seed sensor, the unpaired *t* test demonstrated significantly lower LIs (i.e., less left lateralization) of gamma-1 band coherence between the temporal sensor (seed sensor) and the sensors in the parietal regions in children with ASD. As shown in Fig. 5b, when we used the parietal region as a seed sensor, the unpaired *t* test demonstrated significantly lower LIs (i.e., less left lateralization) of gamma-1 band coherence between the parietal sensor (seed sensor) and the sensors in the temporal regions in children with ASD.

### 3.6 Spontaneous gamma band power and developmental delay

With an alpha level of 0.05, at the risk of a Type I error, unpaired *t* tests demonstrated significantly higher relative power values in the gamma-1 band in the sensors corresponding to the left frontal (*t* = 2.12, *P* < 0.05) and central (*t* = 2.02, *P* < 0.05) areas and in the gamma-2 band in a sensor corresponding to left frontal area (*t* = 2.36, *P* < 0.05) in ASD children compared to TD children.

**Fig. 6:**
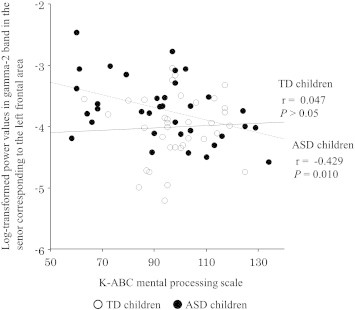
Scatter plot of log-transformed relative power values in the gamma-2 band between the sensor corresponding to the left frontal area and the K-ABC mental processing scale in children with ASD (r = − 0.429, P = 0.010) and TD children (r = 0.047, P > 0.05). ○, TD children (n = 35); ●, ASD children (n = 35). ASD, autism spectrum disorder; TD, typically developing; Solid line, regression line for TD children; Broken line, regression line for ASD children.

To investigate whether these increased gamma band powers in young children with ASD were related to the degree of developmental delay, we calculated Pearson’s correlation coefficients to evaluate the relationships between the relative power values in the gamma bands in which significant differences were found (with an alpha level of 0.05) with two types of general cognitive quotients on the K-ABC (normalized with age) in both young children with ASD and in TD children. The significance level was set at *P* < 0.05. As a result, a significant negative correlation was found only in the young children with ASD between the relative power values in the gamma-2 band left frontal sensor and the K-ABC mental processing scale (*r* = − 0.423, *P* < 0.05) (Fig. 6).

## 4 Discussion

We examined neurophysiological connectivity in young children under conscious measurement conditions and provided initial evidence of aberrant, rightward-lateralized connectivity via gamma oscillation in young children with ASD, including in children aged 3–4 years, which was not demonstrated in our recent study of older children with ASD (5–7 years old) (Kikuchi et al., 2013). To estimate functional brain connectivity, we used MEG not only because it is a less stressful method for young children but also because MEG is superior to EEG, with respect to spatial resolution, for determining ongoing brain activity in sensor space (Hari et al., 1988; Quandt et al., 2012), although MEG and EEG offer the same temporal resolution. The lower spatial resolution of scalp EEG signals is due to spatial blurring at the interface of tissues with different conductances. This limitation cannot be overcome by increasing the density of the EEG electrodes.

Converging evidence suggests that the properties of gamma oscillations are altered in ASD during information processing (Brown et al., 2005; Grice et al., 2001; Orekhova et al., 2007; Perez Velazquez et al., 2009; Stroganova et al., 2012; Sun et al., 2012; Wilson et al., 2007; Wright et al., 2012). In children with ASD (aged 3 to 8 years), a previous EEG study demonstrated that an excess of gamma band oscillations during sustained visual attention is directly related to the degree of developmental delay (Orekhova et al., 2007). Our results also demonstrated that the tendency of excess gamma band oscillations in the frontal area while viewing a video program was related to the degree of developmental delay (Fig. 6). The altered gamma band activity that we observed in young children with ASD may result from disturbances in the GABAergic or glutamatergic mediator systems, which are critically important for generating this type of oscillation (Sohal et al., 2009) and suggest that changes in the balance of excitation and inhibition may be a pervasive feature of brain dysfunction in ASD starting from a young age (Rubenstein and Merzenich, 2003). There are two possibilities to explain the altered gamma band oscillations in young children with ASD. The first explanation is that we observed an ongoing aberrant balance of excitation and inhibition in the young children with ASD (i.e., increased gamma power in spontaneous brain activity). The second explanation is that an aberrant balance of excitation and inhibition during the fetal period results in aberrant prenatal and perinatal development, which leads to lasting alterations in neural networks. Gamma-frequency oscillations between neural networks are essential for cortical information processing (Jensen et al., 2007; Wang, 2010), and oscillations in the posterior regions are believed to represent ongoing cognitive processes during visual perception and attention (Beauchamp et al., 2012; Brookes et al., 2005; Gregoriou et al., 2009; Keil et al., 1999; Privman et al., 2011; Tallon-Baudry and Bertrand, 1999; Zion-Golumbic and Bentin, 2007). The results of the present study demonstrate altered brain networks, as evidenced by gamma band oscillation, during the viewing of a video program in young children with ASD. In fact, atypical visual perception and cognition (Keita et al., 2011; Klin et al., 2009) and altered gamma band activity during visual perception (Brown et al., 2005; Grice et al., 2001; Stroganova et al., 2012; Sun et al., 2012; Wright et al., 2012) have previously been reported in ASD subjects. Intriguingly, a recent study provides direct evidence that altered visual performance is accompanied by altered gamma band activity in ASD subjects (Sun et al., 2012). Furthermore, previous studies using other methods (e.g., magnetic resonance imaging) also support our results (i.e., a rightward functional network in ASD), demonstrating that individuals with ASD tend to make use of the right hemisphere during various conditions, as described in the following paragraphs.

Recent studies have demonstrated rightward (or less leftward) functional connectivity in adults with ASD both during rest (Monk et al., 2009) and during working memory tasks (Koshino et al., 2005) using functional connectivity magnetic resonance imaging (fcMRI) methods. Although fcMRI has not previously been performed in young children with ASD, a recent study suggested an atypical developmental trajectory in older children with ASD (8–12 years old). The results demonstrated that functional connectivity in the right hemisphere decreased with age in these children, although the ASD children did not show significant differences compared to the TD children (Lee et al., 2009). This suggests the intriguing possibility that younger ASD children (i.e., < 8 years old) have higher degrees of right intrahemispheric connectivity.

From the perspective of cortical responses to stimuli in young ASD children, we refer to a fMRI study that suggested aberrant right lateralization by demonstrating a trend toward greater recruitment of the right hemispheric regions during speech stimulation (Redcay and Courchesne, 2008).

From an anatomical perspective, we refer to a recent study that used voxel-based morphometric analysis to demonstrate that young ASD children (approximately 2 years of age) have a larger volume of white matter in the right hemisphere than TD children (Hoeft et al., 2011). These observations support our results of rightward intrahemispheric connectivity. Although there have been only a few previous reports of young children with ASD, our results, in conjunction with other findings, suggest that aberrant rightward brain function is a hallmark of ASD present at very young ages.

Comparisons of intrahemispheric coherences with an alpha level of 0.05, risking a Type I error, showed a number of significantly higher intrahemispheric coherences in ASD children (Fig. 2). This may indicate intrinsic overconnectivity in ASD children, as described in recent studies (Monk et al., 2009; Noonan et al., 2009; Shih et al., 2011), as opposed to other studies suggesting underconnectivity in ASD (Coben et al., 2008; Just et al., 2004, 2007; Kana et al., 2006, 2007; Villalobos et al., 2005). A recent review of fcMRI studies suggested that particular choices of MRI analyses of functional connectivity (e.g., low-pass filtering and whole-brain analysis) would demonstrate overconnectivity in intrinsic brain activity in ASD (Muller et al., 2011). The overconnectivity of spontaneous brain activity observed in the present and previous studies may represent disruptions of local functional differentiation and the associated sculpting of distributed specialized networks in ASD children. In addition, from an anatomical perspective, our results in young ASD children are consistent with previous findings from diffusion tensor imaging (DTI) studies in which elevated fractional anisotropy values (i.e., elevated white matter integrity) in toddlers with ASD were reported (Ben Bashat et al., 2007; Weinstein et al., 2011). In contrast, studies of older ASD children and adults have indicated reduced fractional anisotropy (i.e., reduced white matter integrity) (Alexander et al., 2007; Barnea-Goraly et al., 2004). Aberrant higher spontaneous brain connectivity may be a temporal hallmark of ASD at very young ages.

To gain insight into the development of brain network dysfunction in ASD, it is necessary to study the pathophysiology of young children with ASD, as an analysis of the brains of older ASD children may not reveal the origin of these developmental processes. For example, although brain size in autism is equivalent to or slightly smaller than the normal average at birth, abnormally accelerated brain growth is observed during the first few years of life (Courchesne et al., 2011; Redcay and Courchesne, 2005). In later childhood, brain growth slows or is arrested. This abnormal age-related trajectory of brain growth strongly suggests that brain functions measured in young children with ASD may demonstrate robust pathophysiological abnormalities. From an anatomical perspective, we refer to previous studies in young children with ASD (Ben Bashat et al., 2007; Hoeft et al., 2011; Sundaram et al., 2008) that demonstrate accelerated abnormal maturation of the white matter. However, it is challenging to measure functional brain connectivity in very young ASD children under conscious conditions, as it is difficult to maintain their cooperation during the functional measurements. In fact, the present study is the first to focus on functional brain connectivity under conscious conditions in young children with ASD, and we demonstrated aberrant lateralization in young children with ASD, including in children aged 3–4 years, which was not demonstrated in our recent study of 5- to 7-year-old children with ASD (Kikuchi et al., 2013).

A critical limitation should be taken into account when considering the results of the coherence analysis performed here. Assessment of coherence (i.e., phase correlation extracted from a Fourier transform) determines the degree of phase lag consistencies between activities recorded at different sensors. However, coherence between a pair of sensors can increase not only because there are two distinct sources, providing more phase-locked activity, but also because a single source can generate a signal that reaches multiple sensors (Srinivasan et al., 2007). In the present study, to reduce the possibility of the latter, we employed sparse alignment of the MEG sensors (e.g., the distance between the temporal and parietal regions was 120 mm in each hemisphere). As magnetic field strength diminishes with the square of the distance from the source, there must be a strong source of gamma oscillation if only one source influenced the coherence between the temporal and parietal areas. However, in the present study, power analyses of the temporal and parietal areas failed to demonstrate any significant differences between groups in the gamma-1 band. Therefore, our conclusion that intrahemispheric phase-locking between two distinct brain regions (i.e., the temporal and parietal areas) is aberrantly right-lateralized in young children with ASD is valid.

This study has several other limitations. First, there is a possibility that the differences in measured coherence may relate to the different temporospatial properties of the video stimuli during the periods corresponding to the selected artifact-free segments. In addition, we did not evaluate the degree to which the subjects attended to the auditory or visual stimuli. The children with ASD might have attended to the visual information, rather than to the narrative auditory information. Differences in these modality-dependent preferences may be reflected in the functional brain connectivity. Second, the reliability of coherence in the gamma band is reported to be lower than that of the alpha band (Gudmundsson et al., 2007), and these coherences could be significantly affected by the mental state of the subjects (Gasser et al., 1987). Further studies that employ longer periods of measurement and attention-controlled conditions will provide more reliable evidence, although these conditions will be difficult to achieve in conscious young children. Third, given that young children were examined in the present study, we were unable to obtain structural brain information on which to superimpose the coordinate systems of the source-estimated MEG signals, as described in a previous study of adults with ASD (Honaga et al., 2010). This limitation was encountered because it is troublesome, especially when studying young children, to perform additional MR imaging. We therefore performed sensor-level analysis as opposed to voxel-based analysis. However, future studies using child-friendly, open-type MRI devices and source-space coherence analysis with beam-forming methods (Sekihara et al., 2011), which have become a popular method of estimating functional connectivity based on MEG/EEG, will enable us to investigate the source level brain network in young children under conscious conditions.

## Figures and Tables

**Table 1:** Demographic characteristics of all participants.

Group	ASD children	TD children	*t* value
Number of subjects	35	35	
Age in month (range)	65.8 (40–93)	64.7 (39–85)	0.41 (n.s.)
Gender (M/F)	29/6	29/6	
K-ABC mental processing scale (± SD)	92.7(± 21.0)	98.5(± 13.5)	1.38 (n.s.)
K-ABC achievement scale (± SD)	93.9 (± 22.20)	98.5(± 13.27)	1.05 (n.s.)

K-ABC, Kaufman Assessment Battery for Children; TD, typically developing; ASD, Autism Spectrum Disorder; n.s., no significant difference (i.e., unpaired *t* test between two groups, *P* > 0.05).

## Appendix A Supplementary data

Table S1 The t-values of intrahemispheric coherence for each hemisphere between ASD (n = 35) and TD (n = 35) children.

Table S2 The t-values in LIs for intrahemispheric coherence between ASD (n = 35) and TD (n = 35) children.

Table S3 The t-values of relative power values for each hemisphere between ASD (n = 35) and TD (n = 35) children.
