# Supplementary material for: Altered brain connectivity in 3-to 7-year-old children with autism spectrum disorder
Source: Neuroimage Clin. 2013 Mar 19;2:394–401. doi: 10.1016/j.nicl.2013.03.003 (PMC3777701; doi:10.1016/j.nicl.2013.03.003)
Supplement: Table S1 — The t-values of intrahemispheric coherence for each hemisphere between ASD (n = 35) and TD (n = 35) children. [file mmc1.doc]

NeuroImage CLINICAL

Title: Altered brain connectivity in 3- to 7-year-old children with autism spectrum disorder.

Supplemental information

**Results**

**Table S1. The *t*-values of intrahemispheric coherence for each hemisphere between ASD (*n* = 35) and TD (*n* = 35) children**

| Left | Delta | Theta-1 | Theta-2 | Alpha-1 | Alpha-2 | Beta-1 | Beta-2 | Gamma-1 | Gamma-2 |
| --- | --- | --- | --- | --- | --- | --- | --- | --- | --- |
| T - O | 1.55 | 0.58 | 1.61 | 0.12 | 1.51 | 1.58 | 0.72 | 1.23 | 2.24* |
| P - O | 0.96 | 0.77 | 1.47 | 1.92 | 2.37* | 1.64 | 1.66 | 1.50 | 1.36 |
| C - O | 1.58 | 1.24 | 1.00 | 1.31 | 1.37 | 1.28 | 1.71 | 1.51 | 1.68 |
| F - O | 1.39 | 0.77 | 1.23 | 1.25 | 1.40 | 1.54 | 0.74 | 1.30 | 1.53 |
| P - T | 1.41 | 1.65 | 1.51 | 0.10 | 1.64 | 1.59 | 1.71 | 1.23 | 2.45* |
| C - T | 1.95 | 1.29 | 2.11* | 0.12 | 1.59 | 0.74 | 1.93 | 1.70 | 1.72 |
| F - T | 0.78 | 1.54 | 0.35 | -0.39 | 1.20 | 1.68 | 0.89 | 2.09* | 1.21 |
| C - P | 0.20 | 1.32 | 0.79 | -0.50 | 0.97 | 0.36 | -0.15 | 1.06 | 1.45 |
| F - P | 0.94 | 2.38* | 1.62 | -0.70 | 1.75 | 1.60 | 1.85 | 1.34 | 1.75 |
| C - F | 1.06 | 1.46 | 1.16 | -0.46 | 1.96 | 1.11 | 0.19 | 1.79 | 1.47 |
| Right | Delta | Theta-1 | Theta-2 | Alpha-1 | Alpha-2 | Beta-1 | Beta-2 | Gamma-1 | Gamma-2 |
| T - O | 1.75 | 1.55 | 1.28 | 1.72 | 0.82 | 1.70 | -0.15 | 2.70* | 2.12* |
| P - O | 1.13 | 1.96 | 1.74 | 1.71 | 1.31 | 1.58 | 0.86 | 1.90 | 2.79* |
| C - O | 1.27 | 1.64 | 1.05 | 0.08 | 1.07 | 0.98 | 0.92 | 1.37 | 0.96 |
| F - O | 2.65* | 1.94 | 1.33 | -0.28 | 2.10* | 1.41 | 0.78 | 1.35 | 1.62 |
| P - T | 2.10* | 1.48 | 0.85 | 1.03 | 1.72 | 1.50 | 1.94 | 2.85* | 2.46* |
| C - T | 2.75* | 1.44 | 0.66 | -0.26 | 1.26 | 0.73 | 1.72 | 1.18 | 1.48 |
| F - T | 2.40* | 0.74 | -0.54 | -0.71 | 0.59 | -0.45 | -0.90 | 1.34 | 0.98 |
| C - P | -0.30 | 0.66 | 0.50 | -0.44 | 2.06* | 0.91 | 0.51 | 1.76 | 1.75 |
| F - P | 2.94* | 0.75 | 0.01 | 0.55 | 1.97 | 0.99 | 0.71 | 1.19 | 1.74 |
| C - F | 1.47 | 0.60 | -1.54 | -1.05 | 1.54 | 0.01 | -0.42 | 1.03 | 0.98 |

Positive values represent greater left lateralization in the ASD than in the TD group. ASD, autism spectrum disorder; TD, typically developing; F, frontal; C, central; P, parietal; O, occipital; T, temporal. *, *P* < 0.05. There were no significant differences if we used the Bonferroni adjustment (*P* < 0.00056). Please note that significance at an alpha level of 0.05 involves the risk of a Type I error.

Table S2. The *t*-values in LIs for intrahemispheric coherence between ASD (*n* = 35) and TD (*n* = 35) children

| LI | Delta | Theta-1 | Theta-2 | Alpha-1 | Alpha-2 | Beta-1 | Beta-2 | Gamma-1 | Gamma-2 |
| --- | --- | --- | --- | --- | --- | --- | --- | --- | --- |
| T - O | 0.00 | -1.07 | 0.57 | -0.36 | 0.42 | 1.33 | -0.02 | -0.87 | -0.98 |
| P - O | -0.19 | -1.00 | -0.08 | -0.14 | 1.98 | -0.33 | 1.19 | -0.95 | -2.35* |
| C - O | 0.34 | -0.44 | -0.16 | 0.82 | 0.59 | 0.60 | 1.20 | 0.30 | 1.50 |
| F - O | -1.10 | -1.70 | 0.46 | 0.89 | -0.09 | 0.59 | -0.39 | -0.22 | -0.82 |
| P - T | -0.47 | 0.00 | 0.98 | -0.90 | 0.63 | -0.02 | -0.12 | -3.63** | -0.59 |
| C - T | -0.09 | 0.25 | 0.69 | -0.65 | 0.11 | -1.14 | 0.37 | 0.97 | 0.48 |
| F - T | -1.54 | 0.40 | -0.17 | 0.40 | 0.76 | 1.08 | 0.45 | 1.13 | 0.25 |
| C - P | 0.92 | 0.94 | 0.78 | 0.13 | -1.48 | -0.69 | -0.75 | -0.68 | -0.38 |
| F - P | -1.81 | 1.33 | 1.50 | -0.20 | 0.49 | 1.20 | 2.08* | 0.44 | 0.11 |
| C - F | -0.55 | 0.86 | 1.78 | -0.35 | 1.18 | 2.17* | 0.75 | 1.36 | 0.88 |

Positive values represent greater left lateralization in the ASD than in the TD group. ASD, autism spectrum disorder; TD, typically developed; LI, laterality index; F, frontal; C, central; P, parietal; O, occipital; T, temporal. *, *P* < 0.05; **, *P* < 0.00056. Please note that significance at an alpha level of 0.05 involves the risk of a Type I error.

Table S3. The *t*-values of relative power values for each hemisphere between ASD (*n* = 35) and TD (*n* = 35) children

| Left | Delta | Theta-1 | Theta-2 | Alpha-1 | Alpha-2 | Beta-1 | Beta-2 | Gamma-1 | Gamma-2 |
| --- | --- | --- | --- | --- | --- | --- | --- | --- | --- |
| F | 1.38 | -0.65 | -0.63 | 0.02 | 0.74 | 0.10 | -0.26 | 2.12* | 2.36* |
| C | 1.81 | 1.74 | -1.17 | -1.45 | 1.51 | 0.64 | 1.74 | 2.02* | 1.13 |
| T | -0.41 | -2.28* | -1.74 | -0.08 | 1.24 | 1.40 | 1.23 | 1.94 | 1.42 |
| P | 0.64 | 0.60 | -1.51 | -0.23 | -0.02 | 0.11 | 0.76 | 1.91 | 1.71 |
| O | -0.66 | 0.73 | -1.86 | -0.71 | 0.66 | 1.31 | 0.86 | 0.38 | 0.47 |
| Right | Delta | Theta-1 | Theta-2 | Alpha-1 | Alpha-2 | Beta-1 | Beta-2 | Gamma-1 | Gamma-2 |
| F | 1.64 | 1.07 | -0.22 | -1.35 | 1.64 | 0.31 | -0.49 | 1.24 | 1.98 |
| C | 0.99 | 0.28 | -0.85 | -0.45 | 1.31 | 0.87 | 0.51 | 0.80 | 0.58 |
| T | 0.57 | 0.02 | -1.21 | 0.38 | 2.15* | -1.23 | -0.68 | 0.19 | 1.11 |
| P | -0.01 | 0.38 | -0.38 | 0.37 | -0.71 | -0.21 | -0.06 | 1.19 | 0.29 |
| O | 0.27 | 0.99 | -0.29 | -0.97 | -0.94 | 0.67 | 0.82 | 0.58 | 0.65 |

Positive values represent greater power values in the ASD than in the TD group. ASD, autism spectrum disorder; TD, typically developed; F, frontal; C, central; P, parietal; O, occipital; T, temporal. *, *P* < 0.05. There were no significant differences if we used the Bonferroni adjustment (*P* < 0.00111). Please note that significance at an alpha level of 0.05 involves the risk of a Type I error.
